# Hyponatremia is associated with unfavorable outcomes after reperfusion treatment in acute ischemic stroke

**DOI:** 10.1111/ene.16156

**Published:** 2023-11-28

**Authors:** Anissa Pelouto, Jorieke Reimer, Ewout J. Hoorn, Adrienne A. M. Zandbergen, Heleen M. den Hertog

**Affiliations:** ^1^ Department of Internal Medicine, Erasmus Medical Center University Medical Center Rotterdam Rotterdam the Netherlands; ^2^ Department of Neurology Medisch Spectrum Twente Enschede the Netherlands; ^3^ Department of Neurology Isala Hospital Zwolle the Netherlands

**Keywords:** endovascular thrombectomy, hyponatremia, intravenous thrombolysis, prognosis, stroke

## Abstract

**Background and purpose:**

In patients with acute ischemic stroke, hyponatremia (plasma sodium < 136 mmol/L) is common and associated with unfavorable outcomes. However, data are limited for patients who underwent intravenous thrombolysis (IVT) and/or endovascular thrombectomy (EVT). Therefore, our aim was to assess the impact of hyponatremia on postreperfusion outcomes.

**Methods:**

We analyzed data of consecutive patients who presented with acute ischemic stroke and were treated with IVT and/or EVT at Isala Hospital, the Netherlands, in 2019 and 2020. The primary outcome measure was the adjusted common odds ratio (acOR) for a worse modified Rankin Scale (mRS) score at 3‐month follow‐up. Secondary outcomes included symptomatic intracranial hemorrhage, in‐hospital mortality, infarct core, and penumbra volumes.

**Results:**

Of the 680 patients (median age = 73 years, 49% female, median National Institutes of Health Stroke Scale = 5), 430 patients (63%) were treated with IVT, 120 patients (18%) with EVT, and 130 patients (19%) with both. Ninety‐two patients (14%) were hyponatremic on admission. Hyponatremia was associated with a worse mRS score at 3 months (acOR = 1.76, 95% confidence interval [CI] = 1.12–2.76) and in‐hospital mortality (aOR = 2.39, 95% CI = 1.23–4.67), but not with symptomatic intracranial hemorrhage (OR = 1.17, 95% CI = 0.39–3.47). Hyponatremia was also associated with a larger core (17.2 mL, 95% CI = 2.9–31.5) and core to penumbra ratio (55.0%, 95% CI = 7.1–102.9).

**Conclusions:**

Admission hyponatremia in patients with acute ischemic stroke treated with IVT and/or EVT was independently associated with unfavorable postreperfusion outcomes, a larger infarct core, and a larger core to penumbra ratio. Future studies should address whether correction of hyponatremia improves the prognosis.

## INTRODUCTION

In acute ischemic stroke, an arterial occlusion leads to oxygen depletion in the affected brain tissue, which is initially still potentially salvageable (penumbra), but as time progresses gradually converts into irreversibly infarcted tissue (infarct core) [1]. Acute reperfusion therapy is aimed at salvaging viable tissue by preventing conversion of penumbra into infarct core [2]. Tissue viability can be assessed with computed tomographic perfusion (CTP), which allows for distinction and quantification of ischemic core and penumbra volume [3]. To date, at least one quarter of the patients in the Netherlands who present with acute ischemic stroke undergo reperfusion therapy [4], and this number is expected to rise [5].

Previous studies have shown that up to 19% of patients with acute ischemic stroke present with hyponatremia (plasma sodium < 136 mmol/L) [6], which has been consistently related to increased disability and mortality [6, 7, 8]. These studies, however, were conducted in general cohorts of unselected patients with acute ischemic stroke. Therefore, they did not capture the specific impact of hyponatremia on outcomes after reperfusion therapy with intravenous thrombolysis (IVT) and/or endovascular thrombectomy (EVT).

We hypothesized that hyponatremia may contribute to a poor functional outcome (modified Rankin scale [mRS] score >2 at 3 months), despite a successful procedure (modified Thrombolysis in Cerebral Infarction [mTICI] score ≥ 2b) in two ways. First, in the (sub)acute phase of ischemic stroke, hyponatremia can worsen cerebral edema, which may further harm the already injured brain tissue [6]. Second, hyponatremia can compromise the ability to tolerate reperfusion therapy through exacerbation of ischemia–reperfusion injury [9].

Given that the use of reperfusion therapy for acute ischemic stroke is increasing and hyponatremia is both common and potentially modifiable, it is relevant to study its prognostic value for postreperfusion outcomes. Therefore, we aimed to study the association of admission hyponatremia with in‐hospital outcomes and postdischarge functional outcomes after IVT and/or EVT. In addition, we assessed CTP‐derived ischemic core and penumbra volumes to explore differences between patients with and without hyponatremia.

## METHODS

### Study design and data collection

This single‐center, retrospective cohort study was performed at Isala Hospital, the Netherlands. Medical ethics approval for this study (#20230110) was obtained from Isala Hospital's institutional review board, and informed consent was waived due to the observational and retrospective design of the study. We included all consecutive patients aged 18 years and older with a clinical diagnosis of acute ischemic stroke who underwent IVT with alteplase and/or EVT between 1 January 2019 and 31 December 2020. Patients who underwent IVT within 4.5 h and/or EVT within 6 h after stroke onset as well as those treated outside these time windows were included in the analysis. Data collection was based on automated extraction using the validated Clinical Data Collector (version 2.0; CTcue, Amsterdam, the Netherlands) [10]. Collected variables included demographics, stroke severity assessed with the National Institutes of Health Stroke Scale (NIHSS), cardiovascular history and risk factors, vital and laboratory parameters, stroke onset to treatment time, and mTICI score. Hyperglycemia was defined as plasma glucose > 7.8 mmol/L [11]. Medical history for determination of Charlson Comorbidity Index (CCI) [12] and prestroke activities of daily living (ADL) dependency were collected. The CCI is a weighted index to classify comorbidities and has been extensively validated in hospitalized patients [13]. Commonly used drugs associated with hyponatremia were recorded, including thiazide diuretics, serotonin reuptake inhibitors, and antiepileptic drugs [14].

### Assessment of plasma sodium

Plasma sodium was measured as part of routine laboratory workup at initial presentation in the Emergency Department. Plasma sodium was measured with ion‐selective electrodes using a Cobas 8000 ISE Analyzer (Roche Diagnostics, Indianapolis, IN, USA). Hyponatremia was defined as a plasma sodium concentration of <136 mmol/L [15].

### CTP imaging

CTP images were analyzed by a trained observer using Philips IntelliSpace Portal 9.0, Brain CT Perfusion Package (Royal Philips Healthcare, Best, the Netherlands). Ischemic core was defined as a mean transit time (MTT) 50% higher than that of the contralateral hemisphere (relative MTT > 1.50) and a cerebral blood volume (CBV) < 2.0 mL/100 g. Ischemic penumbra was defined as relative MTT >1.50 and a CBV > 2.0 mL/100 g. Ischemic core and penumbra volumes were recorded in milliliters, and the core to penumbra ratio was calculated as percentage by dividing the core by the penumbra volume, multiplied by 100.

### Outcome measures

The primary outcome was mRS score at 3 months. The secondary outcomes included poor functional outcome or death (mRS > 2) at 3 months and in‐hospital outcomes including successful recanalization, symptomatic intracranial hemorrhage, and mortality. Successful recanalization was considered mTICI score ≥ 2b on angiography [16]. Symptomatic intracranial hemorrhage was defined as computed tomography‐confirmed intracranial hemorrhage with corresponding clinical deterioration during hospitalization. Other outcome measures included CTP‐derived ischemic core volume, penumbra volume, and core to penumbra ratio.

### Statistical analyses

Baseline characteristics were compared between hyponatremic and nonhyponatremic patients on admission. Normally distributed continuous data are presented as mean ± SD and nonparametric data as median with interquartile range (IQR). Categorical data were analyzed with the chi‐squared test and continuous nonparametric data with the Mann–Whitney *U*‐test. The primary outcome measure was the adjusted common odds ratio (acOR) for a shift toward a worse mRS at 3 months, estimated with a generalized multivariable ordinal regression/partial proportional model. The secondary outcomes were analyzed with multivariable logistic regression and reported as adjusted odds ratio (aOR). The multivariable model included age, sex, CCI, prestroke ADL dependency, NIHSS on admission, and history of hypertension. Hyperglycemia was added to the second model, because previous studies reported that admission hyperglycemia is a risk factor for poor outcomes after reperfusion therapy [17, 18]. Because high glucose levels can induce translocational (hypertonic) hyponatremia [15], we also performed a sensitivity analysis with hyponatremia based on glucose‐corrected plasma sodium. In the case of hyperglycemia, the corresponding plasma sodium was corrected for the blood glucose value using the Katz formula [19]. We also performed a sensitivity analysis excluding patients who presented with hypernatremia (plasma sodium > 145 mmol/L). Furthermore, we performed a predefined subgroup analysis excluding patients treated in the late window (>4.5 h for IVT and >6 h for EVT). We also performed a predefined stratification analysis according to reperfusion therapy: IVT versus EVT(+IVT). The EVT(+IVT) group included patients who were treated with EVT only and those who were treated with both IVT and EVT. The association between hyponatremia and CTP‐derived parameters was analyzed with univariable and multivariable linear regression analyses. The multivariable model included age, sex, NIHSS on admission, and hyperglycemia [20]. Multicollinearity in all multivariable models was ruled out. Missingness for all covariates was ≤2%, except for mRS at 3 months (16.6%) and onset‐to‐treatment time (2.8%). Missing values were not imputed. All tests were two‐sided, and *p* < 0.05 was considered statistically significant. Statistical analyses were performed using SPSS version 28.01.1, with the exception of the generalized ordered logistic regression analyses, which were performed with Stata SE version 17.0.

## RESULTS

### Patient characteristics

A total of 726 patients underwent IVT and/or EVT, of whom 680 patients met the inclusion criteria (Figure S1). Four hundred thirty patients were treated with IVT (63%), 120 patients (18%) were treated with EVT, and 130 patients (19%) were treated with both IVT and EVT. The median admission plasma sodium was 139 mmol/L (IQR = 137–141 mmol/L; Figure S1). Ninety‐two patients (14%) were hyponatremic. The majority of the hyponatremic patients (90%) had mild hyponatremia (130–135 mmol/L). Patients with hyponatremia were older and had a higher CCI than patients without hyponatremia (Table 1). In addition, patients with hyponatremia more often were hyperglycemic, more often had a history of hypertension, and more often used diuretics including thiazides. The onset‐to‐treatment time and proportion of patients treated in the late window were similar between patients with and without hyponatremia.

**TABLE 1:** Baseline characteristics.

Characteristic	Hyponatremia, *n* = 92	No hyponatremia, *n* = 588	*p*
Age, years, median (IQR)	77 (69–84)	73 (64–81)	0.001
Female, *n* (%)	51 (55)	283 (48)	0.19
Laboratory parameters, median (IQR)			
Plasma sodium, mmol/L	134 (131–135)	140 (138–141)	<0.001
Plasma glucose, mmol/L	7.1 (6.1–9.2)	6.8 (5.9–8.5)	0.12
Hyperglycemia, *n* (%)	40 (44)	193 (33)	0.046
Plasma potassium, mmol/L	4.2 (3.9–4.5)	4.2 (3.9–4.4)	0.59
eGFR, mL/min/1.73 m^2^	72 (58–86)	71 (56–84)	0.70
Vital parameters, median (IQR)			
Systolic blood pressure, mmHg	151 (129–174)	157 (138–175)	0.25
Diastolic blood pressure, mmHg	87 (70–100)	89 (78–101)	0.10
Heart rate, bpm	80 (71–94)	81 (69–93)	0.69
Temperature, °C	36.9 (36.6–37.3)	37.0 (36.6–37.4)	0.29
Medical history, *n* (%)			
Hypertension	70 (76)	365 (62)	0.009
Hypercholesterolemia	66 (71)	451 (77)	0.29
Atrial fibrillation	20 (22)	137 (23)	0.74
De novo	8 (9)	67 (11)	0.44
Diabetes mellitus	18 (20)	128 (22)	0.62
CCI, median (IQR)	5 (4–6)	4 (3–6)	<0.001
Prestroke ADL independent	82 (89)	504 (88)	0.75
Current smoking	13 (14)	114 (20)	0.22
Prestroke medication use, *n* (%)			
Antihypertensive medication	71 (77)	364 (62)	0.005
Diuretics	36 (39)	159 (27)	0.02
Thiazide diuretics	24 (26)	86 (15)	0.006
Antidepressants	8 (9)	53 (9)	0.92
Antiepileptic drugs	6 (7)	20 (3)	0.15
NIHSS on admission, median (IQR)	6 (4–13)	5 (3–11)	0.05
Reperfusion therapy, *n* (%)			
IVT	61 (66)	369 (63)	0.51
Onset–treatment time, min, median (IQR)	130 (82–191)	124 (85–191)	0.74
EVT	15 (16)	105 (18)	0.72
Onset–treatment time, min, median (IQR)	195 (130–568)	289 (138–624)	0.76
Both	16 (17)	114 (19)	0.65
Onset–treatment time for IVT, min, median (IQR)	100 (64–115)	120 (72–245)	0.09
Onset–treatment time for EVT, min, median (IQR)	191 (123–265)	188 (135–351)	0.46

Abbreviations: ADL, activities of daily living; CCI, Charlson Comorbidity Index; eGFR, estimated glomerular filtration rate; EVT, endovascular thrombectomy; IQR, interquartile range; IVT, intravenous thrombolysis; NIHSS, National Institutes of Health Stroke Scale.

### Postreperfusion outcomes

**FIGURE 1:**
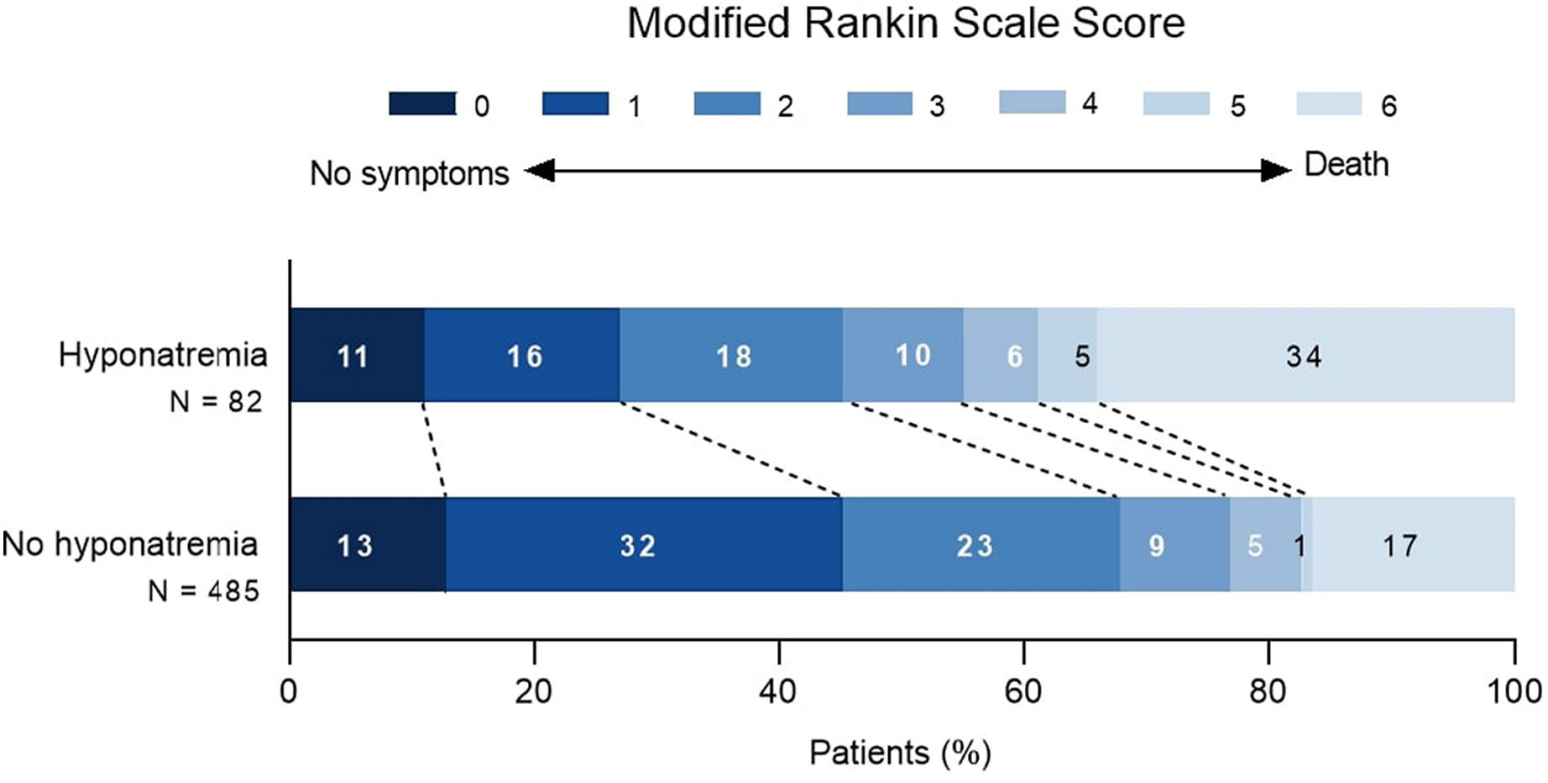
Distribution of the modified Rankin Scale scores at 3 months among patients with and without hyponatremia on admission. Shown is the distribution of scores on the modified Rankin scale. Scores range from 0 (no symptoms) to 6 (death). There was a significant difference between the hyponatremia group and the no hyponatremia group in the overall distribution of scores in an analysis based on multivariable ordinal regression (common odds ratio = 1.76, 95% confidence interval = 1.12–2.76).

Patients with hyponatremia more often had poor functional outcome at 3 months compared to patients without hyponatremia (55% vs. 32%, *p* < 0.001; Table 2). In the multivariable analysis, hyponatremia was independently associated with a shift toward a worse mRS at 3 months (acOR = 1.76, 95% confidence interval [CI] = 1.12–2.76; Table 3, Figure 1). The effect varied across the cutoff values of the mRS in the adjusted models (Table S1). Hyponatremia was also independently associated with in‐hospital mortality (aOR = 2.39, 95% CI = 1.23–4.67; Table 3). Sensitivity analysis with hyponatremia based on glucose‐corrected plasma sodium showed similar results (Table S2). Also, the sensitivity analysis excluding the three patients with hypernatremia showed similar results (data not shown). In a subgroup analysis (*n* = 547) excluding patients treated in the late window, hyponatremia was still independently associated with a shift toward a worse mRS and poor functional outcome at 3 months (Table S3). Furthermore, there was a significant interaction between admission hyponatremia and hyperglycemia (adjusted *p* = 0.007; Table S4). This implies that patients who were both hyponatremic and hyperglycemic on admission showed a stronger shift toward a worse mRS at 3 months (acOR = 2.54, 95% CI = 1.29–4.98). Stratification analysis according to treatment group (IVT vs. EVT[+IVT]) showed that hyponatremia was associated with a shift toward a worse mRS at 3 months in both subgroups. In the EVT(+IVT) subgroup, however, the association lost statistical significance in the multivariable models (acOR = 1.99, 95% CI = 0.79–5.02). For in‐hospital mortality, the association in the EVT(+IVT) subgroup did remain statistically significant in the adjusted models (aOR = 2.64, 95% CI = 1.07–6.52; Table S5). Successful recanalization after EVT, occurrence of a symptomatic intracranial hemorrhage, and discharge destinations did not differ significantly between patients with and without hyponatremia (Table 2). Last, the association of all covariates with poor functional outcome at 3 months and in‐hospital mortality are shown in Table S6.

**TABLE 2:** Frequency of the primary and secondary outcomes among patients with and without admission hyponatremia.

Outcome	Hyponatremia, *n* = 92	No hyponatremia, *n* = 588	*p*
Primary outcome measure, *n* (%)			
mRS score at 3 months, median (IQR)	3 (1–6)	2 (1–3)	<0.001
Secondary outcome measures, *n* (%)			
Poor functional outcome [mRS > 2 at 3 months]	45 (55)	155 (32)	<0.001
Recanalization after EVT [mTICI ≥ 2b]	21 (68)	139 (65)	0.76
Symptomatic intracranial hemorrhage	4 (4)	22 (4)	0.78
In‐hospital mortality	17 (19)	48 (8)	0.002
Discharge destination, *n* (%)			
Home	39 (43)	287 (49)	0.27
Transfer to another hospital	7 (8)	42 (7)	0.86
Rehabilitation facility	19 (21)	163 (28)	0.16
Nursing home	8 (9)	36 (6)	0.34
Hospice	1 (1)	9 (2)	0.75

Abbreviations: EVT, endovascular thrombectomy; IQR, interquartile range; mRS, modified Rankin Scale; mTICI, modified Thrombolysis in Cerebral Infarction.

**TABLE 3:** Association of admission hyponatremia with outcomes after reperfusion therapy.

Outcome	Unadjusted cOR (95% CI)	Model 1 acOR (95% CI)	Model 2 acOR (95% CI)
Primary outcome measure			
mRS score at 3 months	2.35 (1.53–3.61)	1.76 (1.12–2.77)	1.76 (1.12–2.76)
Secondary outcome measures			
Poor functional outcome [mRS > 2 at 3 months], *n* = 200	2.59 (1.61–4.16)	1.99 (1.16–3.42)	1.96 (1.14–3.38)
Recanalization after EVT, [mTICI ≥ 2b], *n* = 160	1.13 (0.51–2.53)	1.10 (0.48–2.51)	1.08 (0.47–2.48)
Symptomatic intracranial hemorrhage, *n* = 26	1.17 (0.39–3.47)		
In‐hospital mortality, *n* = 65	2.55 (1.39–4.66)	2.51 (1.30–4.87)	2.39 (1.23–4.67)

*Note*: Model 1 is adjusted for age, sex, Charlson Comorbidity Index, prestroke activities of daily living dependency, National Institutes of Health Stroke Scale on admission, and history of hypertension. Model 2 is adjusted for factors in model 1 + hyperglycemia on admission.

Abbreviations: acOR, adjusted common odds ratio; CI, confidence interval; cOR, common odds ratio; EVT, endovascular thrombectomy; mRS, modified Rankin Scale; mTICI, modified Thrombolysis in Cerebral Infarction.

### CTP‐derived outcomes

CTP imaging was available in a subset of 143 patients (21%) of the cohort. Of this subset, 17 patients (12%) were hyponatremic. The onset‐to‐imaging times were similar between patients with and without hyponatremia (Table S7). Hyponatremia was independently associated with a larger core volume (17.2 mL, 95% CI = 2.9–31.5) and a higher core to penumbra ratio (55.0%, 95% CI = 7.1–102.9; Table 4). After excluding patients who underwent CTP later than 6 h after stroke onset, we found similar results (Table S8). A sensitivity analysis with hyponatremia based on glucose‐corrected plasma sodium also showed similar results (Table S9). Finally, the association of all covariates with the CTP‐derived parameters is shown in Table S10.

**TABLE 4:** Association of admission hyponatremia with computed tomographic perfusion‐derived parameters.

Parameter	Unadjusted B (95% CI)	Adjusted B (95% CI)^a^
Perfusion deficit, mL	13.7 (−20.1 to 47.5)	8.6 (−23.0 to 40.3)
Core volume, mL	19.8 (14.6 to 25.0)	17.2 (2.9 to 31.5)
Penumbra volume, mL	−10.1 (−38.3 to 18.1)	−8.6 (−36.4 to 19.2)
Core to penumbra ratio, %	63.0 (16.9 to 109.2)	55.0 (7.1 to 102.9)

*Note*: Results are based on multivariable linear regression analysis. B's reflect the change in the outcome variables (perfusion deficit, core volume, penumbra volume, and core‐to‐penumbra ratio) when the exposure (hyponatremia) is present.

Abbreviation: CI, confidence interval.

^a^ Adjusted for age, sex, National Institutes of Health Stroke Scale on admission, and hyperglycemia.

## DISCUSSION

In the present study, we assessed the prognostic impact of hyponatremia on in‐hospital and postdischarge outcomes in patients with acute ischemic stroke who were treated with IVT and/or EVT. Our data show that hyponatremia was independently associated with a twofold increased risk for in‐hospital mortality and poor functional outcome at 3 months. Furthermore, in the subset of patients with available CTP data, hyponatremia was independently associated with a larger core volume and core to penumbra ratio.

One previous study has assessed the association of hyponatremia with functional outcomes in patients with acute ischemic stroke treated with IVT. In this recent study, He et al. found that hyponatremia was associated with poor postthrombolysis outcome (mRS > 2) at 3 months (aOR = 1.65, 95% CI = 1.01–2.68) [21]. In another study in patients with acute ischemic stroke who were treated with EVT, higher admission plasma sodium was associated with lower 3‐month mRS scores (aOR = 0.94, 95% CI = 0.89–0.99) [22]. In our study, we confirm the association of hyponatremia with poor functional outcome at 3 months in a large combined IVT/EVT cohort, which also included patients treated in a late window. Also, we identified an association between hyponatremia and in‐hospital mortality in both the IVT and EVT(+IVT) subgroups.

Hyponatremia is commonly associated with higher mortality in hospitalized patients [23]. It remains, however, unclear whether hyponatremia by itself can contribute directly to mortality or is a marker for the severity of the underlying disease [24]. Although our study is unable to address causality, it is conceivable that hyponatremia—at least in part—contributes to morbidity and mortality by negatively affecting the disease course in acute ischemic stroke. For example, He et al. showed that hyponatremia was associated with more postthrombolysis hemorrhagic transformations, although they did not report whether this resulted in more neurological deterioration or mortality [21]. In our study, no association between hyponatremia and symptomatic intracerebral hemorrhages in patients treated with either IVT or EVT(+IVT) was identified.

Pathophysiologically, the association of hyponatremia with poor outcomes in acute ischemic stroke may be explained by a number of mechanisms. First, in the acute phase of ischemic stroke hyponatremia may exacerbate the formation of ionic edema [25]. Next, the ischemia‐induced altered permeability of the blood–brain barrier leads to further disruption of the intracerebral electrolyte homeostasis [26]. As a result, the brain may become more at risk for fluid shifts. Even small increments in percentage of brain water content reflect great changes in brain swelling [27]. Brain swelling can cause further ischemia through increased intracranial pressure and decreased capillary perfusion [28]. Based on our CTP data, hyponatremia was associated with a larger ischemic core volume and core to penumbra ratio. This may be an indication of increased infarct growth in patients with hyponatremia. Accelerated conversion of penumbra into irreversibly infarcted tissue prior to treatment would reduce the potential treatment benefit of reperfusion therapy [29]. Furthermore, an additional hypothesis is that hyponatremia activates the reverse mode of the Na^+^/Ca^2+^ exchanger, resulting in increased intracellular Ca^2+^ levels and production of reactive oxygen species [9, 30]. These changes reduce the recoverability of penumbral tissue. After inducing an episode of ischemia–reperfusion injury in experimental studies, hyponatremic rats were shown to have significantly larger myocardial infarct sizes compared to the normonatremic controls [9]. Thus, hyponatremia may lead to undesired effects at both the immediate pre‐ and postreperfusion stage and ultimately result in poor outcomes.

Interestingly, we found that patients who were both hyponatremic and hyperglycemic had the highest risk of a worse mRS at 3 months. Hyperglycemia has been shown to promote downstream microvascular thromboinflammation and decreased fibrinolytic ability of alteplase [17, 31]. Combined with the deleterious effects of hyponatremia, this may synergistically diminish the likelihood of achieving good outcomes after reperfusion therapy.

Based on the available data, it is difficult to ascertain the etiology of hyponatremia, which would require information on urinary osmolality and sodium. Similarly, we cannot determine whether hyponatremia was acute or chronic. Acute hyponatremia may occur as a result of increased vasopressin secretion secondary to acute stroke [6], although it is also conceivable that many patients had preexisting chronic hyponatremia due to comorbidities or medication. For example, chronic hyponatremia may have been caused by thiazide diuretics, which were more commonly used by the hyponatremic patients in this study.

Strengths of the present study include the large, diverse cohort of patients treated with IVT, EVT, or both in a real‐world setting, which enhances the generalizability of our findings. Another strength is the availability of corresponding CTP data in a subset of the patients, which allowed quantification of core and penumbra volumes to identify a radiological substrate for the clinical findings. To our knowledge, this is the first study to analyze the relationship of hyponatremia with CTP‐derived ischemic core and penumbra volumes. We also performed sensitivity analyses based on glucose‐corrected plasma sodium in addition to the main analysis, which controlled for hyperglycemia. The results of these analyses corroborate an effect of hyponatremia independent of hyperglycemia.

Our study also has a number of limitations. First, mRS scores at 3 months were missing in 16.6% of the patients. Because loss to follow‐up was similar in patients with and without hyponatremia, and there were no differences in discharge destination, we believe that the missing data did not introduce bias. Second, lack of statistical power in the subgroup analyses may have prevented us from detecting a significant association with infrequent complications such as symptomatic intracerebral hemorrhage. Third, due to the lack of repeated plasma sodium measurements, it remains unknown whether hyponatremia was transient or persisted throughout hospitalization.

In conclusion, admission hyponatremia in patients treated with IVT and/or EVT for acute ischemic stroke is associated with in‐hospital mortality and poor functional outcome at 3 months. Our findings encourage further research in this area. For example, post hoc analysis of data from randomized controlled trials on reperfusion therapy could assess whether hyponatremia confers treatment effect modification. Furthermore, prospective studies should address whether correction of hyponatremia improves postreperfusion outcomes. Based on previous ischemia–reperfusion data [9, 30], we suggest experimental studies for studying the effects of rapid correction of hyponatremia using intravenous bolus hypertonic (3%) saline therapy.

## AUTHOR CONTRIBUTIONS

**Anissa Pelouto:** Conceptualization; writing – original draft; formal analysis; data curation; project administration; writing – review and editing. **Jorieke Reimer:** Data curation; software; writing – review and editing. **Ewout J. Hoorn:** Supervision; writing – review and editing; conceptualization. **Adrienne A. M. Zandbergen:** Conceptualization; writing – review and editing; supervision. **Heleen M. Den Hertog:** Conceptualization; writing – review and editing; methodology; supervision.

## CONFLICT OF INTEREST STATEMENT

None of the authors has any conflict of interest to disclose.

## Supporting information


TABLE S1
TABLE S2
TABLE S3
TABLE S4
TABLE S5
TABLE S6
TABLE S7
TABLE S8
TABLE S9
TABLE S10
FIGURE S1
FIGURE S2

## Data Availability

The data that support the findings of this study are available from the corresponding author upon reasonable request.
