# Supplementary material for: Hyponatremia is associated with unfavorable outcomes after reperfusion treatment in acute ischemic stroke
Source: Eur J Neurol. 2023 Nov 28;31(3):e16156. doi: 10.1111/ene.16156 (PMC11236033; doi:10.1111/ene.16156)
Supplement: Supplementary file 1 — TABLE S1 TABLE S2 TABLE S3 TABLE S4 TABLE S5 TABLE S6 TABLE S7 TABLE S8 TABLE S9 TABLE S10 FIGURE S1 FIGURE S2 [file ENE-31-e16156-s001.docx]

**R1, 27-10-2023**

**Supplemental material**

**Hyponatremia is associated with Unfavorable Outcomes after Reperfusion Treatment in Acute Ischemic Stroke**

**Table S1.** Association of admission hyponatremia with shifts on the modified Rankin Scale score at 3 months across all cut-off values.

**Table S2.** Association of admission hyponatremia (glucose corrected) with outcomes after reperfusion therapy.

**Table S3.** Association of admission hyponatremia with outcomes after reperfusion therapy, excluding patients treated in the late window (n=547).

**Table S4.** Interaction between admission hyponatremia and hyperglycemia in multivariable regression analysis.

**Table S5.** Association of admission hyponatremia with outcomes after reperfusion therapy, stratified according to treatment group.

**Table S6.** Association of the covariates with poor functional outcome at 3 months and in-hospital mortality.

**Table S7.** CT-perfusion derived parameters among patients with and without hyponatremia.

**Table S8.** Association of admission hyponatremia with CT-perfusion derived parameters, excluding patients who underwent a CT-perfusion scan > 6 hours after stroke onset.

**Table S9.** Association of admission hyponatremia (glucose corrected) with CT-perfusion derived parameters.

**Table S10.** Association of the covariates with the CT-perfusion derived parameters**.**

**Figure S1**. Inclusion and exclusion flowchart.

**Figure S2.** Distribution of admission plasma sodium.

**Table S1.** Association of admission hyponatremia with shifts on the modified Rankin Scale score (mRS) at 3 months across all cut-off values.

|  | Unadjusted  OR (95% CI) | Model 1  aOR (95% CI) | Model 2  aOR (95% CI) |
| --- | --- | --- | --- |
| **Overall point estimate / common OR** | 2.35 (1.53 – 3.61) | 1.76 (1.12 – 2.77) | 1.76 (1.12 – 2.76) |
| **Across the mRS** |  |  |  |
| mRS 1 – 6 vs 0 | 2.35 (1.53 – 3.61) | 0.88 (0.39 – 1.96) | 0.89 (0.40 – 1.99) |
| mRS 2 – 6 vs 0 – 1 | 2.35 (1.53 – 3.61) | 2.37 (1.33 – 4.23) | 2.42 (1.36 – 4.31) |
| mRS 3 – 6 vs 0 – 2 | 2.35 (1.53 – 3.61) | 3.15 (1.77 – 5.59) | 3.22 (1.81 – 5.73) |
| mRS 4 – 6 vs 0 – 3 | 2.35 (1.53 – 3.61) | 4.63 (2.42 – 8.86) | 5.93 (3.04 – 11.57) |
| mRS 5 – 6 vs 0 – 4 | 2.35 (1.53 – 3.61) | 8.10 (3.79 – 17.30) | 14.85 (6.24 – 35.32) |
| mRS 6 vs 0 – 5 | 2.35 (1.53 – 3.61) | 0.05 (0.01 – 0.17) | 0.01 (0.003 – 0.07) |

Model 1 is adjusted for age, sex, Charlson Comorbidity Index, pre-stroke Activities of Daily Living dependency, National Institute Health Stroke Scale on admission and history of hypertension.

Model 2 is adjusted for factors in model 1 + hyperglycemia on admission.

**Table S2.** Association of admission hyponatremia (glucose corrected) with outcomes after reperfusion therapy.

| **Primary outcome measure** | Unadjusted  cOR (95% CI) | Model 1  acOR (95% CI) |
| --- | --- | --- |
| mRS score at 3 months | 2.18 (1.39 – 3.41) | 1.67 (1.04 – 2.67) |
| **Secondary ordinal outcome measure** | Unadjusted  OR (95% CI) | Model 1  aOR (95% CI) |
| Poor functional outcome,  (mRS >2 at 3 months), n=200 | 2.23 (1.36 – 3.65) | 1.77 (1.01 – 3.11) |
| Recanalization after EVT, (mTICI ≥ 2b), n=160 | 0.83 (0.36 – 1.93) | 0.80 (0.34 – 1.89) |
| Symptomatic intracranial haemorrhage, n=26 | 1.36 (0.46 – 4.06) |  |
| In-hospital mortality, n=65 | 2.25 (1.18 – 4.27) | 2.19 (1.09 – 4.42) |

mRS, modified Rankin Scale; mTICI, modified Thrombolysis In Cerebral Infarction.

Model 1 is adjusted for age, sex, Charlson Comorbidity Index, pre-stroke Activities of Daily Living dependency, National Institute Health Stroke Scale on admission and history of hypertension.

**Table S3.** Association of admission hyponatremia with outcomes after reperfusion therapy, excluding patients treated in the late window (n=547).

| **Primary outcome measure** | Unadjusted  acOR (95% CI) | Model 1  acOR (95% CI) | Model 2  acOR (95% CI) |
| --- | --- | --- | --- |
| mRS score at 3 months | 2.14 (1.34 – 3.44) | 1.65 (1.01 – 2.70) | 1.65 (1.01 – 2.70) |
| **Secondary outcome measures** | Unadjusted  OR (95% CI) | Model 1  aOR (95% CI) | Model 2  aOR (95% CI) |
| Poor functional outcome,  (mRS >2 at 3 months), n=147 | 2.57 (1.52 – 4.33) | 1.96 (1.07 – 3.58) | 1.94 (1.06 – 3.55) |

mRS, modified Rankin Scale; mTICI, modified Thrombolysis In Cerebral Infarction.

Model 1 is adjusted for age, sex, Charlson Comorbidity Index, pre-stroke Activities of Daily Living dependency, National Institute Health Stroke Scale on admission and history of hypertension.

Model 2 is adjusted for factors in model 1 + hyperglycemia on admission.

**Table S4.** Interaction between admission hyponatremia and hyperglycemia for outcomes after reperfusion therapy.

| **Primary outcome measure** | Unadjusted  Interaction, P value | Adjusted*  Interaction, P value |
| --- | --- | --- |
| mRS score at 3 months | <0.001 | 0.007 |
| **Secondary outcome measures** |  |  |
| Poor functional outcome,  (mRS >2 at 3 months), n=200 | 0.04 | 0.07 |
| Recanalization after EVT,  (mTICI ≥ 2b), n=160 | 0.87 | 0.71 |
| Symptomatic intracranial haemorrhage, n=26 | 0.94 |  |
| In-hospital mortality, n=65 | 0.10 | 0.12 |

mRS, modified Rankin Scale; mTICI, modified Thrombolysis In Cerebral Infarction.

* Adjusted for age, sex, Charlson Comorbidity Index, pre-stroke Activities of Daily Living dependency, National Institute Health Stroke Scale on admission and history of hypertension.

**Table S5.** Association of admission hyponatremia with outcomes after reperfusion therapy, stratified according to treatment group.

|  | | Unadjusted  acOR (95% CI) | Model 1  acOR (95% CI) | Model 2  acOR (95% CI) |
| --- | --- | --- | --- | --- |
| **Primary outcome measure** | |  |  |  |
| mRS score at 3 months | IVT,  (n=460) | 2.75 (1.59 – 4.75) | 1.80 (1.02 – 3.18) | 1.81 (1.02 – 3.20) |
|  | EVT(+IVT),  (n=250) | 2.39 (1.11 – 5.15) | 1.98 (0.79 – 4.97) | 1.99 (0.79 – 5.02) |
| **Secondary outcome measures** | | Unadjusted  OR (95% CI) | Model 1  aOR (95% CI) | Model 2  aOR (95% CI) |
| mRS > 2 at 3 months | IVT,  (n=89) | 3.53 (1.94 – 6.41) | 2.24 (1.13 – 4.43) | 2.25 (1.13 – 4.64) |
|  | EVT(+IVT), (n=111) | 2.12 (0.88 – 5.09) | 1.73 (0.67 – 4.43) | 1.64 (0.64 – 4.23) |
| mTICI ≥ 2b | IVT,  N.A. |  |  |  |
|  | EVT(+IVT), (n=160) | 1.12 (0.50 – 2.50) | 1.08 (0.47 – 2.48) | 1.07 (0.47 – 2.46) |
| Symptomatic intracranial haemorrhage | IVT,  (n=15) | 0.42 (0.06 – 3.27) |  |  |
|  | EVT(+IVT),  (n=11) | 2.83 (0.71 – 11.28) |  |  |
| In-hospital mortality | IVT,  (n=19) | 3.86 (1.46 – 10.23) |  |  |
|  | EVT(+IVT),  (n=46) | 2.42 (1.05 – 5.57) | 2.69 (1.10 – 6.59) | 2.64 (1.07 – 6.52) |

mRS, modified Rankin Scale; mTICI, modified Thrombolysis In Cerebral Infarction; IVT, intravenous thrombolysis; EVT, endovascular thrombectomy; N.A., not applicable

Model 1 is adjusted for age, sex, Charlson Comorbidity Index, pre-stroke Activities of Daily Living dependency, National Institute Health Stroke Scale on admission and history of hypertension.

Model 2 is adjusted for factors in model 1 + hyperglycemia on admission.

**Table S6.** Association of all the covariates with poor functional outcome at 3 months and in-hospital mortality.

|  | **Outcomes** (adjusted OR (95% CI)) | |
| --- | --- | --- |
|  | Poor functional outcome  (mRS > 2 at 3 months) | In-hospital mortality |
| Age, per year | 1.05 (1.03 – 1.08) | 1.05 (1.02 – 1.09) |
| Sex, male | 1.24 (0.81 – 1.90) | 2.10 (1.14 – 3.88) |
| Charlson Comorbidity Index | 1.09 (0.97 – 1.22) | 1.03 (0.88 – 1.20) |
| Pre-stroke Activities of Daily Living dependency | 0.67 (0.36 – 1.24) | 1.06 (0.42 – 2.68) |
| National Institute Health Stroke Scale | 1.17 (1.13 – 1.21) | 1.14 (1.09 – 1.19) |
| Hypertension | 1.14 (0.73 – 1.78) | 0.89 (0.48 – 1.66) |
| Hyperglycemia | 1.21 (0.78 – 1.86) | 1.57 (0.88 – 2.81) |
| Hyponatremia | 1.96 (1.14 – 3.38) | 2.39 (1.23 – 4.67) |

Results are based on multivariable logistic regression analysis.

**Table S7.** CT-perfusion derived parameters among patients with and without hyponatremia.

| \|  \| **Hyponatremia**  **(n = 17)** \| **No hyponatremia**  **(n = 125)** \| **P-value** \| \| --- \| --- \| --- \| --- \| \| Onset-imaging time in minutes, median (IQR) \| 121 (58 – 433) \| 126 (71 – 371) \| 0.84 \| \| Perfusion deficit in mL, median (IQR) \| 114 (69 – 141) \| 77 (27 – 146) \| 0.36 \| \| Core volume in mL, median (IQR) \| 28 (7 – 79) \| 9 (3 – 28) \| 0.02 \| \| Penumbra volume in mL, median (IQR) \| 65 (35 – 91) \| 64 (23 – 117) \| 0.83 \| \| Core to penumbra ratio in %, median (IQR) \| 32 (12 – 140) \| 17 (5 – 54) \| 0.06 \| |
| --- | --- | --- | --- | --- | --- | --- | --- | --- | --- | --- | --- | --- | --- | --- | --- | --- | --- | --- | --- | --- | --- | --- | --- | --- |

**Table S8.** Association of admission hyponatremia with CT-perfusion derived parameters, excluding patients who underwent a CT-perfusion scan > 6 hours after stroke onset.

|  | Unadjusted  B (95% CI) | Adjusted*  B (95% CI) |
| --- | --- | --- |
| Perfusion deficit in mL | 15.8 (-25.7 – 57.4) | 2.8 (-35.2 – 40.7) |
| Core volume in mL | 28.7 (9.3 – 48.1) | 19.2 (1.7 – 36.7) |
| Penumbra volume in mL | -12.9 (-46.6 – 20.9) | -16.4 (-49.5 – 16.7) |
| Core to penumbra ratio in % | 80.8 (19.0 – 142.7) | 75.4 (11.9 – 138.9) |

* Adjusted for age, sex, National Institute Health Stroke Scale on admission and hyperglycemia.

**Table S9.** Association of admission hyponatremia (glucose corrected) with CT-perfusion derived parameters.

|  | Unadjusted  B (95% CI) | Adjusted*  B (95% CI) |
| --- | --- | --- |
| Perfusion deficit in mL | 14.5 (-22.3 – 51.3) | 3.1 (-30.7 – 36.9) |
| Core volume in mL | 22.8 (6.5 – 39.2) | 17.7 (2.5 – 32.9) |
| Penumbra volume in mL | -8.3 (-39.1 – 22.4) | -14.6 (-44.6 – 15.5) |
| Core to penumbra ratio in % | 70.1 (19.9 – 120.3) | 71.1 (20.4 – 121.9) |

* Adjusted for age, sex and National Institute Health Stroke Scale on admission.

**Table S10.** Association of all the covariates with the CT-perfusion derived parameters**.**

| Covariate | Outcome: CT-perfusion derived parameters (adjusted B (95% CI)) | | | |
| --- | --- | --- | --- | --- |
|  | Perfusion deficit (mL) | Core  (mL) | Penumbra  (mL) | Core to penumbra ratio (%) |
| Age, per year | -0.4 (-1.1 – 0.4) | -0.02 (-0.4 – 0.3) | -0.4 (-1.0 – 0.3) | 0.4 (-0.8 – 1.5) |
| Sex, male | 12.6 (-7.4 – 32.6) | 7.3 (-1.8 – 16.3) | 5.3 (-12.2 – 22.9) | 32.6 (2.4 – 62.9) |
| NIHSS | 4.5 (3.0 – 6.0) | 1.8 (1.1 – 2.5) | 2.8 (1.4 – 4.1) | 0.04 (-2.2 – 2.3) |
| Hyperglycemia | -20.4 (-42.5 – 1.7) | 3.9 (-6.1 – 13.9) | -24.3 (-43.7; -4.9) | 23.3 (-10.1 – 56.7) |
| Hyponatremia | 8.6 (-23.0 – 40.3) | 17.2 (2.9 – 31.5) | -8.6 (-36.4 – 19.2) | 55.0 (7.1 – 102.9) |

NIHSS, National Institute Health Stroke Scale

Results are based on multivariable linear regression analysis.

**Figure S1**. Inclusion and exclusion flowchart.


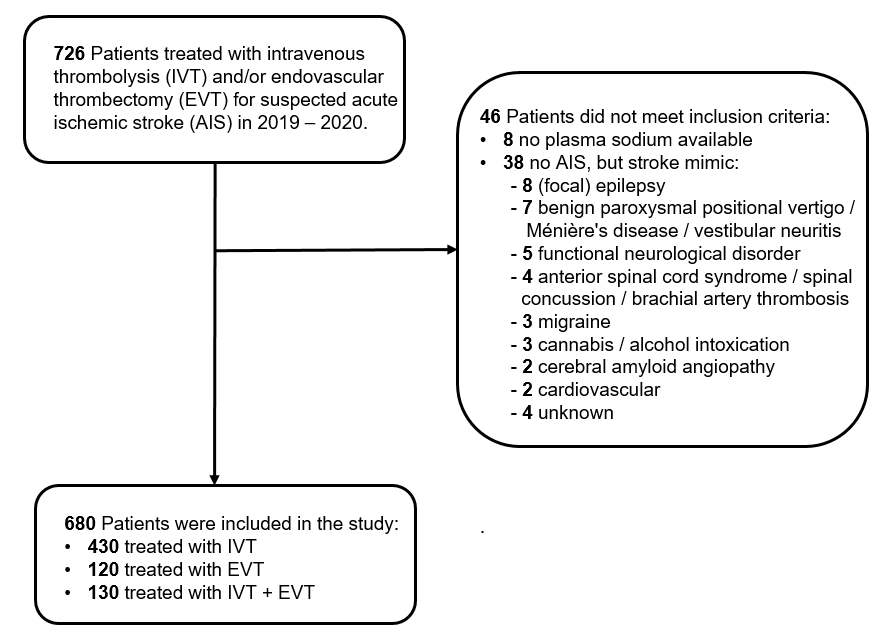


**Figure S2.** Distribution of admission plasma sodium.

**
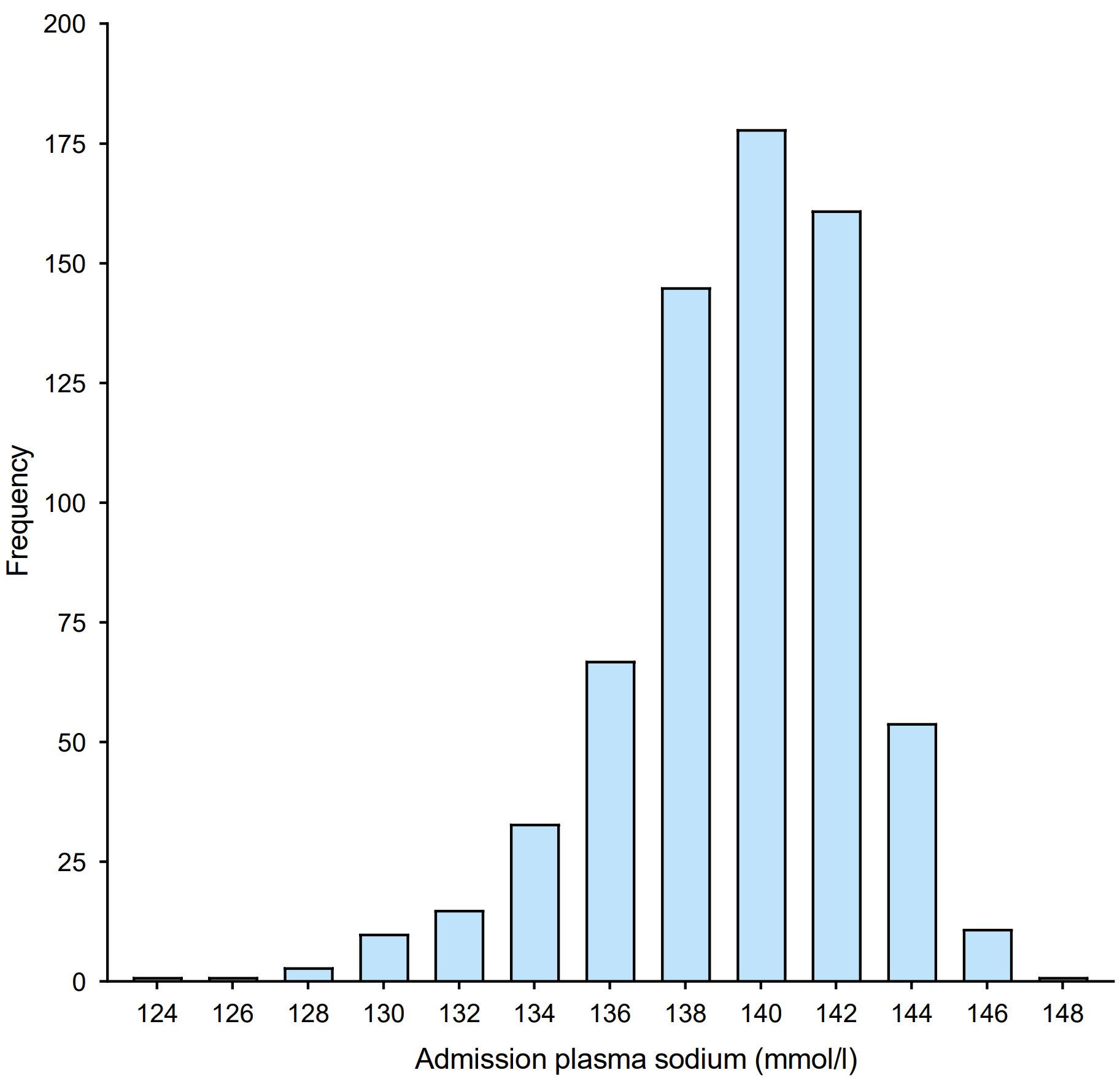
**
